# Medical News and Miscellany

**Published:** 1889-01

**Authors:** 


### Medical News and Miscellany.

THE American Medical Association will meet at Newport, R. I., June 25, 1889.

DR. W. R. WILSON, of Dallas, was married in Texarkana, on January 9th, to Miss Bettie Thomas of that city.

DR. A. B, ASHWORTH, the able editor of the Atlanta Medical and Surgical Journal, has become sole owner of that valuable property.

DR. W. A. HAMMOND opened his sanitarium in Washington on the 10th inst., with one-half his rooms filled with patients. "Just as we expected."

DRS. STINSON & THOMPSON, an old firm of Sherman—have dissolved their co-partnership. Dr. Stinson renews his subsctip-tion to the JOURNAL individually.

STATE HEALTH OFFICER OF TEXAS, Dr. Rutherford has been appointed by Governor Ross, for a second term as State Health Officer, and the appointment will be confirmed by the senate.

APOLOGETIC.—During the greater part of December the Editor of this JOURNAL was sick, and could not give his personal attention to the details of the December number, in conseqnence of which many mortifying errors occurred—especially in the "Contents." We crave indulgence and will try to not get sick any more. *D. V.*

"A DOCTOR FIRED," is the caption of an article in a Memphis paper announcing that at the late meeting of the Tri-State Medical Association held in Memphis, Dr. J. P. McGee was expelled by unanimous vote,—the members rising. Dr. McGee is the ex-professor wbo tried to forge a diploma for the man Mc-Kelsey—an account of which appeared in our October number.

THE VALUE OF AN EYE.—Dr. C. H. Wilkinson, of Galveston, as surgeon of the Santa Fe railroad, treated one of the employes for conjunctivitis, caused, alleged, by a cinder in the eye from a passing train. The sight of one eye was destroyed and the other impaired in consequence of the inflammation. Now the party brings suit against the road and the surgeon for $10,-000. The way of the (surgeon) is hard indeed.

---

THE SUPERINTENDENT TEXAS STATE LUNATIC ASYLUM discharged recenty as "improved," a female patient of twenty years residence in the asylum. Her son was to take her home. On the way to the train, and just after leaving the institute, she died in the hack. Said to have been heart disease. Respectfully submitted without comment; facts learned from the daily papers.

---

DR. T. M. MATTHEWS, of Athens, Texas, was burned out December 4, ult, losing his office, drugs, books and instruments. No insurance. This is hard luck, as it is the second time in five years that the Doctor has suffered by fire,—in the first instance, losing still more heavily. We tender our sincere condolence to the Doctor in his bad luck: But, he is a philosopher, and says very truthfully, "it might be worse."

---

MARRIED.—Our talented and rising young friend, Dr. Eugene A. Neely, of Memphis, Tenn., was married on January 23d inst., to Miss Ida Bruce of that city, With a knowiedge of Dr. Neely's æsthetic taste, we are sure we can congratulate him on his choice; and we are equally sure that we can congratulate the young lady in having secured *a prize* in the lottery matrimonial.

---

JORDAN W. LAEBERT, president of the Lambert Pharmacal Co., of St. Louis, died in that city January 9th, after a lingering illness, nerve exhaustion, said to have been superinduced by a too active and wearing life. Mr. Lambert was one of the most popular and enterprising men of St. Louis, and is a great loss to social and commercial circles. He was well and favorably known to the medical profession.

WE DO OUR OWN SINGING—At the suggestion of a mutual friend we wrote to a certain physician in Texas who had made some reputation as a medical writer, soliciting an occasional contribution to the clinical department of the JOURNAL, The doctor quite misunderstood us; and having a good opinion of his powers, suggested that he might be induced—*for a consideration*—to write the *editorials* for us if we wished it! ! !

We told him a little anecdote, by way of reply, as was the custom of ths "late lamented" Abraham Lincoln, as follows;

"Once upon a time, the choir of a certain village church was practicing over night for services Sunday; a lean specimen of the genus "tramp," with a thin, piping voice, poked his head in at the window, and, in a shrill key asked: "Do you want to hire anybody to sing bass at this church?" The basso, with a voice like distant thunder, rolled his eyes up at him, and in a tone that shook the rafters, said: "Go-to-hell-Dod-dang-you-I-sing-bass-myself! ! !"

That's all we said.

---

IT is understood that the Governor will not recommend anyone for Superintendent of the (Austin) State Lunatic Asylum, but will leave the election of a successor to Dr. Dorsett, to the Board of Managers, which course, we believe, is in conformity to the law. It is generally believed the Board will not reappoint or elect Dr. Dorsett.

---

IMPORTANT (?) IF TRUE.—It is reported that the defunct "*Southern Journal* of *Homeopathy*," whose obituary appears elsewhere, has been purchased by a "Doctor Clifford" of San Antonio, and that it will be issued in that city by the well known printer, Johnson Bros. We never before heard of Dr. Clifford, but he must be a man of wondrous courage to undertake to do what Fisher failed in, and confessed that the "profession" would not support a journal in its interest, for Fisher is a *rustler* from away back yonder, who is "sharp" enough to make a fortune swapping knives,—but failed as a journalist in a bad cause,—hence the undertaking looks like a forlorn hope.

DR. H. D. BRUNS has been appointed Pathologist to the Charity Hospital, New Orleans, vice Dr. H. D. Schmidt, deceased.

---

AN IMPORTANT DECISION AS AFFECTING TEXAS PHYSICIANS.—Our readers will recall the case of Dr. J. B. Fears, of Nacogdoches county, who was summoned as an expert in a case, and made two post mortem examinations; for this service he presented a bill for $20. The county court awarded him $5 in each case, and he appealed. It will be seen by the following official report of the case how Texas courts look upon professional services of skilled surgeons. It is a burning shame; but there seems to be no redress, even the higher courts taking the narrow view that a physician should be paid only laborers wages:

J. B. Fears, vs. Nacogdoches county—appeal from Nacogdoches.

Appellant, a practicing physician, was summoned to attend upon two inquests for the purpose of making a post mortem examination. Being threatened by justice with an attachment, he appeared, made the examination, and subsequently presented an account to the commissioners' court for $20 in each case. The court allowed $5 in each case, and rejected the balance of the account. He sued in the justice court, and on appeal to the district court he had judgment for $10. The county acquiesed in the judgment and he appeals. Held that the county cannot be held liable for such services. A coroner's inquest is a proceeding by and on behalf of the State. The justice and ex-officio coroner who conducts it, though sometimes called a precinct or county officer, is, to all intents and purposes, an officer of the State, and in exercising his functions acts for the State, and not for the county. The legislature has not made this a charge upon the county further than set out in article 1077, et seq., C. C. P.

Whether or not a physician can be compelled to perform such services, is not decided, as that question is not presented. Affirmed. Opinion by Gaines, J.

[This opinion seems to conflict with a recent decision by the court of appeals.—Rep.]

WE ARE PLEASED to welcome to Austin Dr. W. H. Way, late House Surgeon of the Manhattan Eye and Ear Infirmary, New York. Dr. Way has located in Austin for the practice of his specialty, diseases of the eye and ear, and brings the highest testimonials from leading medical men of New York and Georgia, his old home. He has had a fine experience, and has an established reputation as a skillful oculist.

---

HIC JACET.—The *Southern Journal of Homeopathy*, died of inanition, ostensibly, but most likely, it *crowed* itself to death like Robinson's rooster. With its last gasp the plucky little bantam complained that there was "not sufficient *material* in Texas to support a journal," and after a feeble attempt to appear cheerful and resigned, it gave up its little ghost, folded its little wings and departed this life. Seriously and with all due respect, its editor and manager is a man of pluck, energy, enterprise, and of some intelligence, and had these qualites been brought to bear in a more worthy cause, he would doubtless have achieved success,—but—homeopathy! Bah! nonsense, moonshine. Fisher plied it for all it was worth, and with the above result. California is to be blessed with this important individual's presence we believe, henceforth.

---

### ACROSTIC.

EULOGISTIC TRIBUTE IN MEMORY OF

CORNELIUS KALLOCK GREGG, M. D.,

Who died by accident, while testing on his own person, for the benefit of suffering humanity, the effects of a pain annihilating drug.

Entered into rest, at McKinney, Texas, October 13, 1888, aged 30 years. One of the brightest ornaments of the medical profession; an honorable and Christian gentleman.

———He was a beloved physician, a noble martyr brave;
Christ-like, in love for man he died—in love he died to save.
Our dear old bishop's youngest son, C. Kallock Gregg, M. D.—
"Requiem œternum dona iis, Domine."

No racking pain which drug could reach, yet vanquished him in
fight;
E'er pain was felt, to keep it off, he put forth all his might;
Lived he to find out cures for pain, with art of the profession;
In every seat where pain was found, he made it yield possesion.

Until, one day, abnormal pain attacked a patient's breast,—
Suffering, strange, defied known cures—repelled old remedies
test.
Keen science gave no formula to break this new pain's rod,
All human skill was powerless; the doctor sought his God.

Love prompted him—he knelt, and prayed in agonizing cry,
"Lord help me find a remedy; let not my patient die!
Oh ease him dearest Lord and God; show me how I may find
Cure for this moveless, death-like pain! O Lord, illume my
mind!"

Knelt—till God gave to him new light, and this test of his
love to man:
"Go, prove, at the risk of thine own dear life, this one and only
plan:
Risk thine own life,—for with untried drugs thou mayst not risk
thy brother's."
Enrapt, he took it, dose by dose—reeled madly—died for others.

Gone! read some of his notes in scraps writ down—"the drugs
just as I ground them."
"Give 1, 2, 3,"—"the third's too much,"—"effects just as I
found them."
"Memo, my brain on fire! An accident—I trust in Christ's
great worth."
"Death! No; 'tis but my mortal body's death—my soul's im-
mortal birth."

—By a Texas Priest.

---

IT IS CLAIMED that there are imitations on the market of the celebrated Johan Hoff's Malt Extract. Read Tarrant & Co.'s new whole-page advertisement; they are sole agents for the genuine article. Make no mistake in this, it is important.

